# Have recent efforts successfully boosted adolescent and young adult enrollment in cancer clinical trials?

**Published:** 2022-10

**SEPTEMBER 12, 2022** - Analysis indicates that adolescent and young adult patients are participating as a greater proportion of total enrollment after the 2014 formation of the NCI National Clinical Trials Network.

It’s important for adolescents and young adults (AYAs) to participate in cancer clinical trials to ensure adequate opportunities for AYA patients to contribute to, and benefit from, advances in cancer treatment. A recent analysis published by Wiley online in CANCER, a peer-reviewed journal of the American Cancer Society, indicates that such trials have enrolled a greater proportion of newly diagnosed AYA patients in recent years.

The research examined how AYA enrollment changed after 2014, when the National Cancer Institute (NCI), part of the National Institutes of Health, formed the NCI National Clinical Trials Network (NCTN), which includes four medical oncology cooperative groups, one pediatric group, and one international group that work together to coordinate and support cancer clinical trials at more than 2,200 sites across the United States, Canada, and internationally. One of the NCTN’s goals was to increase patient enrollment in trials in rare cancers and from special populations including AYAs.

Investigators identified 304 phase 2, 2/3, and 3 trials led by one of the cooperative groups that were activated from 2004 through 2019, involved therapy for newly diagnosed cancer, and had age eligibility overlapping the AYA range (15-39 years). AYA accrual comprised 9.5% of enrolled patients in pre-NCTN trials compared with 14.0% of patients in post-NCTN trials. The findings indicate that there has been a proportionate increase in AYA participation in cancer clinical trials after the NCTN was formed.

“Though we cannot directly attribute this increase to launch of the NCTN, these results are encouraging and suggest that the NCTN is working to provide clinical trials for newly diagnosed AYA patients and treating oncologists are taking advantage of these opportunities to enroll patients,” said lead author Hari Sankaran, MD, MSc, of the National Cancer Institute.

*Full Citation: “Enrollment of adolescent and young adult patients newly diagnosed with cancer in NCI CTEP-sponsored clinical trials before and after launch of the NCI National Clinical Trials Network.” Hari Sankaran, Shanda R. Finnigan, Lisa M. McShane, Ana F. Best, and Nita L. Seibel. CANCER; Published Online: September 12, 2022 (DOI: 10.1002/cncr.34402).*

*URL Upon Publication: http://doi.wiley.com/10.1002/cncr.34402*

*Copyright © 2021 The Cochrane Collaboration. Published by John Wiley & Sons, Ltd., reproduced with permission.*

